# Molecular neuroscience at its “high”: bibliometric analysis of the most cited papers on endocannabinoid system, cannabis and cannabinoids

**DOI:** 10.1186/s42238-019-0004-y

**Published:** 2019-06-07

**Authors:** Andy Wai Kan Yeung, Nikolay T. Tzvetkov, Nicolas Arkells, Luigi Milella, Adrian M. Stankiewicz, Łukasz Huminiecki, Olaf K. Horbanczuk, Atanas G. Atanasov

**Affiliations:** 1 Oral and Maxillofacial Radiology, Applied Oral Sciences, Faculty of Dentistry, The University of Hong Kong, Hong Kong, China; 2 Institute of Molecular Biology “Roumen Tsanev”, Department of Biochemical Pharmacology and Drug Design, Bulgarian Academy of Sciences, Sofia, Bulgaria; 3 Pharmaceutical Institute, University of Bonn, Bonn, Germany; 4 Wrazel, 49 Discovery, Suite 180, Irvine, CA 92618 USA; 5 Department of Science, University of Basilicata, V.le dell’Ateneo Lucano 10, 85100 Potenza, Italy; 6 The Institute of Genetics and Animal Breeding, Polish Academy of Sciences, Magdalenka, Poland; 7 Faculty of Human Nutrition and Consumer Sciences, Warsaw University of Life Sciences, 02-776 Warsaw, Poland; 8 Department of Pharmacognosy, University of Vienna, Vienna, Austria; 9 GLOBE Program Association (GLOBE-PA), Grandville, MI USA

**Keywords:** Cannabis, Endocannabinoid, Molecular neuroscience, Bibliometrics, Citation analysis, VOSviewer

## Abstract

**Background:**

Cannabis, cannabinoids and endocannabinoids are heavily investigated topics with many articles published every year. We aimed to identify the 100 most cited manuscripts among the vast literature and analyze their contents.

**Methods:**

Web of Science (WoS) Core Collection was searched to identify the 100 most cited relevant manuscripts, which were analyzed with reference to (1) authorship, (2) institution, (3) country, (4) document type, (5) journal, (6) publication year, (7) WoS category, and (8) citation count. Semantic content and citation data of the manuscripts were analyzed with VOSviewer.

**Results:**

The most cited manuscripts were published between 1986 and 2016, with the majority being published in the 2000s (*n* = 51). The number of citations for the top 100 articles ranged from 469 to 3651, with a median citation count of 635.5. The most prolific authors were Vincenzo Di Marzo (*n* = 11) and Daniele Piomelli (*n* = 11). The major contributing countries were USA (*n* = 49), Italy (*n* = 22), UK (*n* = 19), and France (*n* = 11). The most prolific institutions were University of California (*n* = 14), National Research Council of Italy (*n* = 12) and National Institutes of Health USA (*n* = 12). The manuscripts consisted of original articles (*n* = 75), reviews (*n* = 24) and a note (*n* = 1). The most dominant journal was *Nature* (*n* = 15). The major WoS categories associated were Multidisciplinary sciences (*n* = 31), Neurosciences (*n* = 20), Pharmacology / Pharmacy (*n* = 16), and General / Internal Medicine (*n* = 11).

**Conclusions:**

The top-ranked manuscripts among the 100 were concerning analgesia, weight loss, long-term potentiation, depolarization-induced suppression of inhibition, opiates and other topics. Cannabinoid type 1 (CB1) receptor was studied by more of the top 100 papers in comparison to cannabinoid type 2 (CB2) receptor. The most frequently mentioned chemicals in these publications were 2-arachidonoylglycerol, tetrahydrocannabinol, and anandamide. Together, these manuscripts comprise the most highly cited publications in the topic, literally the molecular neuroscience at its “high”.

## Background

*Cannabis sativa* L. (cannabis) has been cultivated by humanity for more than 2000 years (Mercuri et al. 2002). It is estimated that 7.2% of the USA population abused cannabis during their lifetime (Stinson et al. 2006). The main reason for its recreational use is its ability to produce euphoria, or feeling of “high” (Ashton 2001). The euphoriant effect of cannabinoids derived from cannabis is attributed to their interactions with the mammalian endogenous cannabinoid system; stimulation of the endocannabinoid system may also lead to effects such as perceptual alterations, impaired psychomotor performance, and tachycardia (Ashton 2001). On the other hand, endocannabinoids are important lipid messengers that regulate synaptic transmission and neurotransmitter release in the brain (Piomelli 2003).

The endocannabinoid system consists of G protein-coupled cannabinoid receptors, cannabinoid type 1 (CB1) and type 2 (CB2) receptors, and endogenous lipid-based neurotransmitters, known as the endocannabinoids, that target these receptors in central and peripheral nervous systems (Pertwee 2015). The CB1 receptor was discovered by Bonner and his co-workers back in 1990 (Matsuda et al. 1990) and is mainly concentrated in the brain (Pacher et al. 2006), whereas the CB2 receptor was discovered by Munro et al. in 1993 (Munro et al. 1993) and is mainly found in the immune system and also the gastrointestinal system (Pacher and Mechoulam 2011). Research findings have hinted that there could be additional CB receptors; one candidate of such is GPR55, which was discovered by O’Dowd and his co-workers in 1999 (Sawzdargo et al. 1999) and later found to be activated by various cannabinoids (Ryberg et al. 2007). Other candidates may also exist, though there is still to be a consensus (Rodriguez de Fonseca and Schneider 2008).

Meanwhile, there are various endocannabinoids, including the well-known eicosanoids anandamide and 2-arachidonoylglycerol, which are metabolized by fatty acid amide hydrolase and monoacylglycerol lipase respectively (Pertwee 2006). Other chemicals considered to be endocannabinoids included 2-arachidonyl glyceryl ether (Hanuš et al. 2001), *N*-arachidonoyl dopamine (Bisogno et al. 2000), virodhamine (Porter et al. 2002), and lysophosphatidylinositol (Henstridge et al. 2009). With regard to phytocannabinoids, it is believed that the *Cannabis* plant contains over 100 cannabinoids (Aizpurua-Olaizola et al. 2016), including tetrahydrocannabinol (THC), cannabidiol (CBD) and cannabinol (CBN). Some cannabinoids are psychoactive, and some are not; and they often interact with one another by synergism (Russo 2011). There are also diverse synthetic cannabinoids, including nabilone used as an antiemetic and for neuropathic pain (Herman et al. 1979; Toth et al. 2012).

Due to large volume of literature on the topic, the application of bibliometric analysis can facilitate better understanding of the field. Bibliometric analysis encompasses for example surveying journal editorial practice (Yeung 2017), or assessing the publication and citation data of a specific research field (Yeung et al. 2017a). A recent bibliometric analysis of cannabis-related literature investigated six topics involving genetics, biochemistry, and biology (Matielo et al. 2018). We aimed to evaluate the literature from another perspective, by identifying the most impactful manuscripts concerning endocannabinoid, cannabis and cannabinoid. Further in the manuscript we would relate to these manuscripts as cannabis and cannabinoid-related manuscripts. By analyzing the 100 most cited articles, we aimed to provide a quick guide on the most influential research in the field, which can serve as a starting point for fellow researchers to quickly identify the high impact topics, their contributors, and outlining possible future research directions and collaborations.

In the current manuscript we described the 100 most cited cannabis and cannabinoid-related manuscripts and identified the major contributors and research themes. We also analyzed relationships between the citation count of these manuscripts and various bibliometric parameters, such as author number, reference number, and journal impact factor.

## Methods

### Data sources

Bibliometric data was extracted from Web of Science (WoS) Core Collection online database, a multidisciplinary database hosted by Clarivate Analytics. In November 2018, we queried WoS with string: TOPIC = (“endocannabinoid*” OR “cannabi*”). This query returned manuscripts that contain the words “endocannabinoid”, “cannabis”, “cannabinoid” or their derivatives in their title, abstract or keywords. WoS search engine does not distinguish uppercase and lowercase characters, so we did not include the same search words in uppercase. No additional restriction was placed on the search. The authors’ WoS subscription included publications from the year 1956 forward. Therefore, publications published before that year could not be analyzed.

The manuscripts were sorted by descending citation count. Two authors (AWKY and AGA) assessed the titles and abstracts of the manuscripts to exclude irrelevant ones.

Some manuscripts might use terms such as CB1 (cannabinoid receptor type 1 receptor), CB2 and marijuana without mentioning endocannabinoid or cannabis or cannabinoid. These manuscripts were identified by an additional search with string: TOPIC = (“CB1” OR “CB2” OR “marijuana”) NOT TOPIC = (“endocannabinoid*” OR “cannabi*”).

### Data extraction

The 100 most-cited papers were evaluated for: (1) authorship, (2) institution, (3) country, (4) document type, (5) journal, (6) publication year, (7) WoS category, and (8) citation count.

Pearson’s correlation tests were conducted in SPSS 25.0 (IBM, New York, USA) to evaluate if the citation counts were correlated to the number of authors, number of references and 2017 journal impact factor. Correlations showing *p* < 0.05 were considered significant.

### Bubble maps

The VOSviewer software was used to analyze the semantic content of titles, abstracts and keywords, relate them to citation data, and visualize the results as bubble maps (van Eck and Waltman 2009). Each bubble represents a word or phrase. The bubble size indicates the appearance frequency of the term (multiple appearances in one manuscript count as one). The bubble color indicates the mean citation count received by manuscripts containing the term. Two bubbles are closer to each other if the two terms co-appeared in the manuscripts more frequently. Only terms that appeared in at least 5 of the manuscripts were included in the figure.

## Results and discussion

### Major contributors

**Fig. 1:**
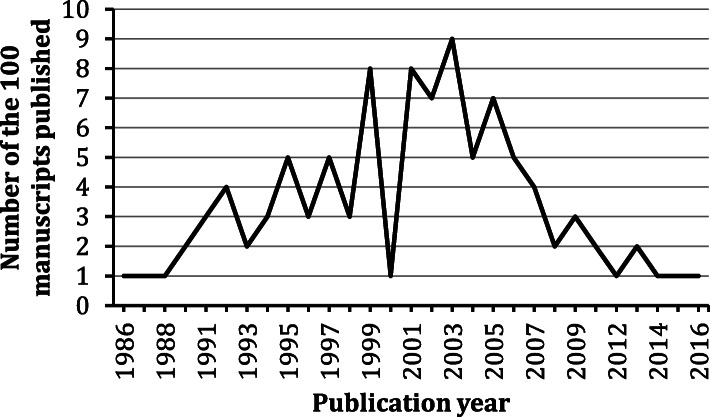
Publication timeline of the 100 most cited cannabis and cannabinoid-related manuscripts

The search resulted in 44,643 manuscripts. The 100 most-cited cannabis manuscripts were all in English. They were published between 1986 and 2016 (Fig. 1). The most prolific authors were Vincenzo Di Marzo (*n* = 11) and Daniele Piomelli (*n* = 11). The major contributing countries were USA (*n* = 49), Italy (*n* = 22), UK (*n* = 19), and France (*n* = 11). The most prolific institutions were University of California (*n* = 14), National Research Council of Italy (*n* = 12) and National Institutes of Health USA (*n* = 12). The manuscripts consisted of original articles (*n* = 75), reviews (*n* = 24) and a note (*n* = 1). The most dominant journal was *Nature* (*n* = 15). The major WoS categories associated with the manuscripts were Multidisciplinary sciences (*n* = 31), Neurosciences (*n* = 20), Pharmacology / Pharmacy (*n* = 16), and General / Internal Medicine (*n* = 11). Consistent to previous surveys on *Cannabis* literature, the majority of the publications focused on the biological/medicinal science instead of plant science (Matielo et al. 2018; Treister-Goltzman et al. 2018). Table 1 lists the top five most prolific authors, institutions, countries, and journals, in terms of their publication count and averaged citations per manuscript. Here, we observed that Italy was behind the USA as the second major contributor, and the Italian roots of the most prolific authors, Vincenzo Di Marzo and Daniele Piomelli. The Italian contribution in the cannabis and cannabinoids research is large compared to research in other related fields, such as ethnopharmacology (1%) (Yeung et al. 2018b), nutraceuticals (2%) (Yeung et al. 2018c), natural products in cancer studies (5.2%) (Yeung et al. 2018a), and neurosciences (5–6.5%) (Yeung 2018; Yeung et al. 2017a; Yeung et al. 2017b). In fact, pollen records has suggested that the central Italy has a long history of cultivating *Cannabis* for more than 2000 years (Mercuri et al. 2002). Moreover, it was estimated that 3.3–5.5 million people in Italy (with a total population of around 61 million) had used cannabis at least once (Farcomeni and Scacciatelli 2013). The abundance of *C. sativa* and the history of its cultivation may partly explain large Italian contribution to the cannabis-related research. It is likely that Italy’s prominence in the field also results from political, regulatory or funding-related factors but analysis of such claims lies beyond scope of this work.

**Table 1:** The major contributors to the 100 most cited cannabis and cannabinoid-related manuscripts

Entity	Publication count	Mean citations per manuscript
Author		
Vincenzo Di Marzo	11	782.5
Daniele Piomelli	11	854.0
Ken Mackie	8	859.8
Roger G. Pertwee	7	1395.7
Raphael Mechoulam	6	1457.0
Institution		
University of California	14	871.4
National Research Council of Italy	12	811.9
National Institutes of Health USA	12	1146.2
University of Washington	8	900.9
University of Aberdeen	7	1395.7
Country		
USA	49	908.2
Italy	22	765.8
UK	19	1049.2
France	11	896.5
Germany	8	715.9
Journal		
Nature	15	1246.1
Proceedings of the National Academy of Sciences of the United States of America	8	792.6
Science	8	1196.5
Lancet	7	686.9
British Journal of Pharmacology	4	560.5

The additional search for identifying manuscripts that mentioned CB1, CB2 or marijuana while omitting terms endocannabinoid, cannabis or cannabinoid returned 11,436 manuscripts. Nineteen of them had citation count of over 469, the citation count of the last manuscript ranked 100th on our list. However, while we focused on molecular biology of the cannabis, most of the additionally identified 19 manuscripts were national surveys or epidemiology studies that targeted drug dependence / substance use / drug addiction, in which marijuana was only one of the many items recorded. The only relevant manuscript was a survey reporting that maternal marijuana use led to decreased infant birth weight and length (Zuckerman et al. 1989).

### Citation counts

The citation counts of the identified 100 papers ranged from 469 to 3651 (mean ± SD: 845.1 ± 546.3, cumulative total citations = 84,510; Table 2). The adjusted citation count (i.e., citation count per year since publication) ranged from 16.6 to 245.5 (mean ± SD: 56.5 ± 38.4, Table 2). Devane et al. (Devane et al. 1992) published the top-ranked manuscript that reported the discovery of anandamide, a natural ligand for the cannabinoid receptors. The total citation count positively correlated with the number of authors (r = 0.232, *p* = 0.023), but not with journal impact factor (r = 0.170, *p* = 0.099), or number of references (r = − 0.044, *p* = 0.668). The adjusted citation count did not correlate with number of authors (r = 0.033, *p* = 0.753), impact factor (r = 0.128, *p* = 0.216) nor number of references (r = 0.038, *p* = 0.714). Meanwhile, the total citation and adjusted citation counts were positively correlated (r = 0.408, *p* < 0.001). These relationships were just partly consistent to the summary of citation analysis literature reported by Hanel and Haase (Hanel and Haase 2017), in which they reported that citation frequency was always positively correlated to impact factor and number of references, while the relationship was mixed for number of authors. The discrepancy here could be explained by the existence of possible research field-specific differences or by the fact that in the current study we analyzed only the 100 most cited manuscripts, so the results of this exploratory correlation analyses may not be the same for the literature body as a whole.

**Table 2:** The list of 100 most cited cannabis and cannabinoid-related manuscripts

Rank	Reference	Impact Factor 2017	Total citation count	Adjusted citation count
1	Devane, W. A., Hanus, L., Breuer, A., Pertwee, R. G., Stevenson, L. A., Griffin, G., ... & Mechoulam, R. (1992). Isolation and structure of a brain constituent that binds to the cannabinoid receptor. *Science*, *258* (5090), 1946–1949.	41.058	3651	140.4
2	Matsuda, L. A., Lolait, S. J., Brownstein, M. J., Young, A. C., & Bonner, T. I. (1990). Structure of a cannabinoid receptor and functional expression of the cloned cDNA. *Nature*, *346* (6284), 561–564.	41.577	3345	119.5
3	Munro, S., Thomas, K. L., & Abu-Shaar, M. (1993). Molecular characterization of a peripheral receptor for cannabinoids. *Nature*, *365* (6441), 61–65.	41.577	3123	124.9
4	Mechoulam, R., Ben-Shabat, S., Hanus, L., Ligumsky, M., Kaminski, N. E., Schatz, A. R., ... & Pertwee, R. G. (1995). Identification of an endogenous 2-monoglyceride, present in canine gut, that binds to cannabinoid receptors. *Biochemical Pharmacology*, *50* (1), 83–90.	4.235	1743	75.8
5	Howlett, A. C., Barth, F., Bonner, T. I., Cabral, G., Casellas, P., Devane, W. A., ... & Mechoulam, R. (2002). International Union of Pharmacology. XXVII. Classification of cannabinoid receptors. *Pharmacological Reviews*, *54* (2), 161–202.	18.964	1651	103.2
6	Devane, W. A., Dysarz, F. 3., Johnson, M. R., Melvin, L. S., & Howlett, A. C. (1988). Determination and characterization of a cannabinoid receptor in rat brain. *Molecular Pharmacology*, *34* (5), 605–613.	3.978	1603	53.4
7	Zygmunt, P. M., Petersson, J., Andersson, D. A., Chuang, H. H., Sørgård, M., Di Marzo, V., ... & Högestätt, E. D. (1999). Vanilloid receptors on sensory nerves mediate the vasodilator action of anandamide. *Nature*, *400* (6743), 452–457.	41.577	1534	80.7
8	Herkenham, M., Lynn, A. B., Little, M. D., Johnson, M. R., Melvin, L. S., De Costa, B. R., & Rice, K. C. (1990). Cannabinoid receptor localization in brain. *Proceedings of the National Academy of Sciences*, *87* (5), 1932–1936.	9.504	1483	53.0
9	Rinaldi-Carmona, M., Barth, F., Héaulme, M., Shire, D., Calandra, B., Congy, C., ... & Ferrara, P. (1994). SR141716A, a potent and selective antagonist of the brain cannabinoid receptor. *FEBS Letters*, *350* (2–3), 240–244.	2.999	1421	59.2
10	Herkenham, M., Lynn, A. B., Johnson, M. R., Melvin, L. S., de Costa, B. R., & Rice, K. C. (1991). Characterization and localization of cannabinoid receptors in rat brain: a quantitative in vitro autoradiographic study. *Journal of Neuroscience*, *11* (2), 563–583.	5.971	1395	51.7
11	Cravatt, B. F., Giang, D. K., Mayfield, S. P., Boger, D. L., Lerner, R. A., & Gilula, N. B. (1996). Molecular characterization of an enzyme that degrades neuromodulatory fatty-acid amides. *Nature*, *384* (6604), 83–87.	41.577	1379	62.7
12	Sugiura, T., Kondo, S., Sukagawa, A., Nakane, S., Shinoda, A., Itoh, K., ... & Waku, K. (1995). 2-Arachidonoylgylcerol: a possible endogenous cannabinoid receptor ligand in brain. *Biochemical and Biophysical Research Communications*, *215* (1), 89–97.	2.559	1347	58.6
13	Mountjoy, K. G., Robbins, L. S., Mortrud, M. T., & Cone, R. D. (1992). The cloning of a family of genes that encode the melanocortin receptors. *Science*, *257* (5074), 1248–1251.	41.058	1282	49.3
14	Piomelli, D. (2003). The molecular logic of endocannabinoid signalling. *Nature Reviews Neuroscience*, *4* (11), 873–884.	32.635	1228	81.9
15	Jordt, S. E., Bautista, D. M., Chuang, H. H., McKemy, D. D., Zygmunt, P. M., Högestätt, E. D., ... & Julius, D. (2004). Mustard oils and cannabinoids excite sensory nerve fibres through the TRP channel ANKTM1. *Nature*, *427* (6971), 260–265.	41.577	1131	80.8
16	Di Marzo, V., Fontana, A., Cadas, H., Schinelli, S., Cimino, G., Schwartz, J. C., & Piomelli, D. (1994). Formation and inactivation of endogenous cannabinoid anandamide in central neurons. *Nature*, *372* (6507), 686–691.	41.577	1115	46.5
17	Moore, T. H., Zammit, S., Lingford-Hughes, A., Barnes, T. R., Jones, P. B., Burke, M., & Lewis, G. (2007). Cannabis use and risk of psychotic or affective mental health outcomes: a systematic review. *The Lancet*, *370* (9584), 319–328.	53.254	1096	99.6
18	Marsicano, G., Wotjak, C. T., Azad, S. C., Bisogno, T., Rammes, G., Cascio, M. G., ... & Di Marzo, V. (2002). The endogenous cannabinoid system controls extinction of aversive memories. *Nature*, *418* (6897), 530–534.	41.577	1075	67.2
19	Tsou, K., Brown, S., Sanudo-Pena, M. C., Mackie, K., & Walker, J. M. (1998). Immunohistochemical distribution of cannabinoid CB1 receptors in the rat central nervous system. *Neuroscience*, *83* (2), 393–411.	3.382	1066	53.3
20	Pacher, P., Bátkai, S., & Kunos, G. (2006). The endocannabinoid system as an emerging target of pharmacotherapy. *Pharmacological Reviews*, *58* (3), 389–462.	18.964	1063	88.6
21	Di Marzo, V., Goparaju, S. K., Wang, L., Liu, J., Bátkai, S., Járai, Z., ... & Kunos, G. (2001). Leptin-regulated endocannabinoids are involved in maintaining food intake. *Nature*, *410* (6830), 822–825.	41.577	1039	61.1
22	Pertwee, R. G. (1997). Pharmacology of cannabinoid CB1 and CB2 receptors. *Pharmacology & Therapeutics*, *74* (2), 129–180.	10.376	1030	49.0
23	Van Gaal, L. F., Rissanen, A. M., Scheen, A. J., Ziegler, O., Rössner, S., & RIO-Europe Study Group. (2005). Effects of the cannabinoid-1 receptor blocker rimonabant on weight reduction and cardiovascular risk factors in overweight patients: 1-year experience from the RIO-Europe study. *The Lancet*, *365* (9468), 1389–1397.	53.254	1024	78.8
24	Kathuria, S., Gaetani, S., Fegley, D., Valiño, F., Duranti, A., Tontini, A., ... & Giustino, A. (2003). Modulation of anxiety through blockade of anandamide hydrolysis. *Nature Medicine*, *9* (1), 76–81.	32.621	1016	67.7
25	Stella, N., Schweitzer, P., & Piomelli, D. (1997). A second endogenous cannabinoid that modulates long-term potentiation. *Nature*, *388* (6644), 773–778.	41.577	1002	47.7
26	Wilson, R. I., & Nicoll, R. A. (2001). Endogenous cannabinoids mediate retrograde signalling at hippocampal synapses. *Nature*, *410* (6828), 588–592.	41.577	996	58.6
27	Després, J. P., Golay, A., & Sjöström, L. (2005). Effects of rimonabant on metabolic risk factors in overweight patients with dyslipidemia. *New England Journal of Medicine*, *353* (20), 2121–2134.	79.26	968	74.5
28	Freund, T. F., Katona, I., & Piomelli, D. (2003). Role of endogenous cannabinoids in synaptic signaling. *Physiological Reviews*, *83* (3), 1017–1066.	24.014	965	64.3
29	Galiègue, S., Mary, S., Marchand, J., Dussossoy, D., Carrière, D., Carayon, P., ... & Casellas, P. (1995). Expression of central and peripheral cannabinoid receptors in human immune tissues and leukocyte subpopulations. *European Journal of Biochemistry*, *232* (1), 54–61.	4.53	963	41.9
30	Ledent, C., Valverde, O., Cossu, G., Petitet, F., Aubert, J. F., Beslot, F., ... & Vassart, G. (1999). Unresponsiveness to cannabinoids and reduced addictive effects of opiates in CB1 receptor knockout mice. *Science*, *283* (5400), 401–404.	41.058	928	48.8
31	Cravatt, B. F., Demarest, K., Patricelli, M. P., Bracey, M. H., Giang, D. K., Martin, B. R., & Lichtman, A. H. (2001). Supersensitivity to anandamide and enhanced endogenous cannabinoid signaling in mice lacking fatty acid amide hydrolase. *Proceedings of the National Academy of Sciences*, *98* (16), 9371–9376.	9.504	905	53.2
32	Everard, A., Belzer, C., Geurts, L., Ouwerkerk, J. P., Druart, C., Bindels, L. B., ... & De Vos, W. M. (2013). Cross-talk between *Akkermansia muciniphila* and intestinal epithelium controls diet-induced obesity. *Proceedings of the National Academy of Sciences*, *110* (22), 9066–9071.	9.504	876	175.2
33	Van Sickle, M. D., Duncan, M., Kingsley, P. J., Mouihate, A., Urbani, P., Mackie, K., ... & Marnett, L. J. (2005). Identification and functional characterization of brainstem cannabinoid CB2 receptors. *Science*, *310* (5746), 329–332.	41.058	861	66.2
34	Pi-Sunyer, F. X., Aronne, L. J., Heshmati, H. M., Devin, J., Rosenstock, J., & RIO-North America Study Group. (2006). Effect of rimonabant, a cannabinoid-1 receptor blocker, on weight and cardiometabolic risk factors in overweight or obese patients: RIO-North America: a randomized controlled trial. *JAMA*, *295* (7), 761–775.	47.661	850	70.8
35	Dinh, T. P., Carpenter, D., Leslie, F. M., Freund, T. F., Katona, I., Sensi, S. L., ... & Piomelli, D. (2002). Brain monoglyceride lipase participating in endocannabinoid inactivation. *Proceedings of the National Academy of Sciences*, *99* (16), 10,819–10,824.	9.504	807	50.4
36	Wilson, R. I., & Nicoll, R. A. (2002). Endocannabinoid signaling in the brain. *Science*, *296* (5568), 678–682.	41.058	799	49.9
37	Cota, D., Marsicano, G., Tschöp, M., Grübler, Y., Flachskamm, C., Schubert, M., ... & Tomassoni, F. (2003). The endogenous cannabinoid system affects energy balance via central orexigenic drive and peripheral lipogenesis. *The Journal of Clinical Investigation*, *112* (3), 423–431.	13.251	786	52.4
38	Caspi, A., Moffitt, T. E., Cannon, M., McClay, J., Murray, R., Harrington, H., ... & Poulton, R. (2005). Moderation of the effect of adolescent-onset cannabis use on adult psychosis by a functional polymorphism in the catechol-O-methyltransferase gene: longitudinal evidence of a gene X environment interaction. *Biological Psychiatry*, *57* (10), 1117–1127.	11.984	784	60.3
39	Calignano, A., La Rana, G., Giuffrida, A., & Piomelli, D. (1998). Control of pain initiation by endogenous cannabinoids. *Nature*, *394* (6690), 277–281.	41.577	782	39.1
40	Katona, I., Sperlágh, B., Sík, A., Käfalvi, A., Vizi, E. S., Mackie, K., & Freund, T. F. (1999). Presynaptically located CB1 cannabinoid receptors regulate GABA release from axon terminals of specific hippocampal interneurons. *Journal of Neuroscience*, *19* (11), 4544–4558.	5.971	761	40.1
41	Tanda, G., Pontieri, F. E., & Di Chiara, G. (1997). Cannabinoid and heroin activation of mesolimbic dopamine transmission by a common μ1 opioid receptor mechanism. *Science*, *276* (5321), 2048–2050.	41.058	731	34.8
42	Marsicano, G., Goodenough, S., Monory, K., Hermann, H., Eder, M., Cannich, A., ... & López-Rodríguez, M. L. (2003). CB1 cannabinoid receptors and on-demand defense against excitotoxicity. *Science*, *302* (5642), 84–88.	41.058	724	48.3
43	Zimmer, A., Zimmer, A. M., Hohmann, A. G., Herkenham, M., & Bonner, T. I. (1999). Increased mortality, hypoactivity, and hypoalgesia in cannabinoid CB1 receptor knockout mice. *Proceedings of the National Academy of Sciences*, *96* (10), 5780–5785.	9.504	721	37.9
44	Osei-Hyiaman, D., DePetrillo, M., Pacher, P., Liu, J., Radaeva, S., Bátkai, S., ... & Kunos, G. (2005). Endocannabinoid activation at hepatic CB 1 receptors stimulates fatty acid synthesis and contributes to diet-induced obesity. *The Journal of Clinical Investigation*, *115* (5), 1298–1305.	13.251	710	54.6
45	Pertwee, R. G., Howlett, A. C., Abood, M. E., Alexander, S. P. H., Di Marzo, V., Elphick, M. R., ... & Mechoulam, R. (2010). International Union of Basic and Clinical Pharmacology. LXXIX. Cannabinoid receptors and their ligands: beyond CB1 and CB2. *Pharmacological Reviews*, *62* (4), 588–631.	18.964	709	88.6
46	Kano, M., Ohno-Shosaku, T., Hashimotodani, Y., Uchigashima, M., & Watanabe, M. (2009). Endocannabinoid-mediated control of synaptic transmission. *Physiological Reviews*, *89* (1), 309–380.	24.014	692	76.9
47	Arseneault, L., Cannon, M., Poulton, R., Murray, R., Caspi, A., & Moffitt, T. E. (2002). Cannabis use in adolescence and risk for adult psychosis: longitudinal prospective study. *BMJ*, *325* (7374), 1212–1213.	23.562	690	43.1
48	Suzuki, A., Josselyn, S. A., Frankland, P. W., Masushige, S., Silva, A. J., & Kida, S. (2004). Memory reconsolidation and extinction have distinct temporal and biochemical signatures. *Journal of Neuroscience*, *24* (20), 4787–4795.	5.971	681	48.6
49	Ryberg, E., Larsson, N., Sjögren, S., Hjorth, S., Hermansson, N. O., Leonova, J., ... & Greasley, P. J. (2007). The orphan receptor GPR55 is a novel cannabinoid receptor. *British Journal of Pharmacology*, *152* (7), 1092–1101.	6.81	679	61.7
50	Finnerup, N. B., Attal, N., Haroutounian, S., McNicol, E., Baron, R., Dworkin, R. H., ... & Kamerman, P. R. (2015). Pharmacotherapy for neuropathic pain in adults: a systematic review and meta-analysis. *The Lancet Neurology*, *14* (2), 162–173.	27.144	639	213.0
51	Andréasson, S., Engström, A., Allebeck, P., & Rydberg, U. (1987). Cannabis and schizophrenia A longitudinal study of Swedish conscripts. *The Lancet*, *330* (8574), 1483–1486.	53.254	632	20.4
52	Wise, R. A. (1996). Neurobiology of addiction. *Current Opinion in Neurobiology*, *6* (2), 243–251.	6.541	623	28.3
53	van Os, J., Kenis, G., & Rutten, B. P. (2010). The environment and schizophrenia. *Nature*, *468* (7321), 203–212.	41.577	615	76.9
54	Bisogno, T., Howell, F., Williams, G., Minassi, A., Cascio, M. G., Ligresti, A., ... & Gangadharan, U. (2003). Cloning of the first sn1-DAG lipases points to the spatial and temporal regulation of endocannabinoid signaling in the brain. *Journal of Cell Biology*, *163* (3), 463–468.	8.784	608	40.5
55	Tchernof, A., & Després, J. P. (2013). Pathophysiology of human visceral obesity: an update. *Physiological Reviews*, *93* (1), 359–404.	24.014	602	120.4
56	Watanabe, H., Vriens, J., Prenen, J., Droogmans, G., Voets, T., & Nilius, B. (2003). Anandamide and arachidonic acid use epoxyeicosatrienoic acids to activate TRPV4 channels. *Nature*, *424* (6947), 434–438.	41.577	597	39.8
57	Beltramo, M., Stella, N., Calignano, A., Lin, S. Y., Makriyannis, A., & Piomelli, D. (1997). Functional role of high-affinity anandamide transport, as revealed by selective inhibition. *Science*, *277* (5329), 1094–1097.	41.058	595	28.3
58	Millan, M. J. (2003). The neurobiology and control of anxious states. *Progress in Neurobiology*, *70* (2), 83–244.	14.163	581	38.7
59	Di Marzo, V., Bifulco, M., & De Petrocellis, L. (2004). The endocannabinoid system and its therapeutic exploitation. *Nature Reviews Drug Discovery*, *3* (9), 771–784.	50.167	580	41.4
60	Volkow, N. D., Baler, R. D., Compton, W. M., & Weiss, S. R. (2014). Adverse health effects of marijuana use. *New England Journal of Medicine*, *370* (23), 2219–2227.	79.26	577	144.3
61	Christensen, R., Kristensen, P. K., Bartels, E. M., Bliddal, H., & Astrup, A. (2007). Efficacy and safety of the weight-loss drug rimonabant: a meta-analysis of randomised trials. *The Lancet*, *370* (9600), 1706–1713.	53.254	568	51.6
62	Felder, C. C., Joyce, K. E., Briley, E. M., Mansouri, J., Mackie, K., Blond, O., ... & Mitchell, R. L. (1995). Comparison of the pharmacology and signal transduction of the human cannabinoid CB1 and CB2 receptors. *Molecular Pharmacology*, *48* (3), 443–450.	3.978	568	24.7
63	Wise, R. A. (1996). Addictive drugs and brain stimulation reward. *Annual Review of Neuroscience*, *19* (1), 319–340.	14.675	567	25.8
64	Deutsch, D. G., & Chin, S. A. (1993). Enzymatic synthesis and degradation of anandamide, a cannabinoid receptor agonist. *Biochemical Pharmacology*, *46* (5), 791–796.	4.235	567	22.7
65	Kreitzer, A. C., & Regehr, W. G. (2001). Retrograde inhibition of presynaptic calcium influx by endogenous cannabinoids at excitatory synapses onto Purkinje cells. *Neuron*, *29* (3), 717–727.	14.319	564	33.2
66	Ohno-Shosaku, T., Maejima, T., & Kano, M. (2001). Endogenous cannabinoids mediate retrograde signals from depolarized postsynaptic neurons to presynaptic terminals. *Neuron*, *29* (3), 729–738.	14.319	560	32.9
67	Blankman, J. L., Simon, G. M., & Cravatt, B. F. (2007). A comprehensive profile of brain enzymes that hydrolyze the endocannabinoid 2-arachidonoylglycerol. *Chemistry & Biology*, *14* (12), 1347–1356.	5.915	556	50.5
68	Giuffrida, A., Parsons, L. H., Kerr, T. M., De Fonseca, F. R., Navarro, M., & Piomelli, D. (1999). Dopamine activation of endogenous cannabinoid signaling in dorsal striatum. *Nature Neuroscience*, *2* (4), 358–363.	19.912	553	29.1
69	Mackie, K., & Hille, B. (1992). Cannabinoids inhibit N-type calcium channels in neuroblastoma-glioma cells. *Proceedings of the National Academy of Sciences*, *89* (9), 3825–3829.	9.504	551	21.2
70	Smart, D., Gunthorpe, M. J., Jerman, J. C., Nasir, S., Gray, J., Muir, A. I., ... & Davis, J. B. (2000). The endogenous lipid anandamide is a full agonist at the human vanilloid receptor (hVR1). *British Journal of Pharmacology*, *129* (2), 227–230.	6.81	548	30.4
71	Arseneault, L., Cannon, M., Witton, J., & Murray, R. M. (2004). Causal association between cannabis and psychosis: examination of the evidence. *The British Journal of Psychiatry*, *184* (2), 110–117.	5.867	547	39.1
72	Rinaldi-Carmona, M., Barth, F., Millan, J., Derocq, J. M., Casellas, P., Congy, C., ... & Portier, M. (1998). SR 144528, the first potent and selective antagonist of the CB2 cannabinoid receptor. *Journal of Pharmacology and Experimental Therapeutics*, *284* (2), 644–650.	3.706	536	26.8
73	Long, J. Z., Li, W., Booker, L., Burston, J. J., Kinsey, S. G., Schlosburg, J. E., ... & Lichtman, A. H. (2009). Selective blockade of 2-arachidonoylglycerol hydrolysis produces cannabinoid behavioral effects. *Nature Chemical Biology*, *5* (1), 37–44.	13.843	533	59.2
74	Dewey, W. L. (1986). Cannabinoid pharmacology. *Pharmacological Reviews*, *38* (2), 151–178.	18.964	530	16.6
75	Ameri, A. (1999). The effects of cannabinoids on the brain. *Progress in Neurobiology*, *58* (4), 315–348.	14.163	520	27.4
76	Hesselbrock, M., Easton, C., Bucholz, K. K., Schuckit, M., & Hesselbrock, V. (1999). A validity study of the SSAGA-a comparison with the SCAN. *Addiction*, *94* (9), 1361–1370.	6.048	519	27.3
77	Glass, M., Faull, R. L. M., & Dragunow, M. (1997). Cannabinoid receptors in the human brain: a detailed anatomical and quantitative autoradiographic study in the fetal, neonatal and adult human brain. *Neuroscience*, *77* (2), 299–318.	3.382	519	24.7
78	Pertwee, R. G. (2008). The diverse CB1 and CB2 receptor pharmacology of three plant cannabinoids: Δ9-tetrahydrocannabinol, cannabidiol and Δ9-tetrahydrocannabivarin. *British Journal of Pharmacology*, *153* (2), 199–215.	6.81	512	51.2
79	Pagotto, U., Marsicano, G., Cota, D., Lutz, B., & Pasquali, R. (2005). The emerging role of the endocannabinoid system in endocrine regulation and energy balance. *Endocrine Reviews*, *27* (1), 73–100.	15.545	512	42.7
80	Breslau, N., Kilbey, M. M., & Andreski, P. (1991). Nicotine dependence, major depression, and anxiety in young adults. *Archives of General Psychiatry*, *48* (12), 1069–1074.	16.642	512	19.0
81	Scheen, A. J., Finer, N., Hollander, P., Jensen, M. D., Van Gaal, L. F., & RIO-Diabetes Study Group. (2006). Efficacy and tolerability of rimonabant in overweight or obese patients with type 2 diabetes: a randomised controlled study. *The Lancet*, *368* (9548), 1660–1672.	53.254	507	42.3
82	Marsicano, G., & Lutz, B. (1999). Expression of the cannabinoid receptor CB1 in distinct neuronal subpopulations in the adult mouse forebrain. *European Journal of Neuroscience*, *11* (12), 4213–4225.	2.832	505	26.6
83	Mailleux, P., & Vanderhaeghen, J. J. (1992). Distribution of neuronal cannabinoid receptor in the adult rat brain: a comparative receptor binding radioautography and in situ hybridization histochemistry. *Neuroscience*, *48* (3), 655–668.	3.382	504	19.4
84	Van Os, J., Bak, M., Hanssen, M., Bijl, R. V., De Graaf, R., & Verdoux, H. (2002). Cannabis use and psychosis: a longitudinal population-based study. *American Journal of Epidemiology*, *156* (4), 319–327.	4.322	503	31.4
85	Kirkham, T. C., Williams, C. M., Fezza, F., & Marzo, V. D. (2002). Endocannabinoid levels in rat limbic forebrain and hypothalamus in relation to fasting, feeding and satiation: stimulation of eating by 2-arachidonoyl glycerol. *British Journal of Pharmacology*, *136* (4), 550–557.	6.81	502	31.4
86	Hanuš, L., Abu-Lafi, S., Fride, E., Breuer, A., Vogel, Z., Shalev, D. E., ... & Mechoulam, R. (2001). 2-Arachidonyl glyceryl ether, an endogenous agonist of the cannabinoid CB1 receptor. *Proceedings of the National Academy of Sciences*, *98* (7), 3662–3665.	9.504	499	29.4
87	Facci, L., Dal Toso, R., Romanello, S., Buriani, A., Skaper, S. D., & Leon, A. (1995). Mast cells express a peripheral cannabinoid receptor with differential sensitivity to anandamide and palmitoylethanolamide. *Proceedings of the National Academy of Sciences*, *92* (8), 3376–3380.	9.504	499	21.7
88	Gerard, C. M., Mollereau, C., Vassart, G., & Parmentier, M. (1991). Molecular cloning of a human cannabinoid receptor which is also expressed in testis. *Biochemical Journal*, *279* (1), 129–134.	3.857	497	18.4
89	Di Marzo, V. (2008). Targeting the endocannabinoid system: to enhance or reduce? *Nature Reviews Drug Discovery*, *7* (5), 438–455.	50.167	494	49.4
90	Degenhardt, L., & Hall, W. (2012). Extent of illicit drug use and dependence, and their contribution to the global burden of disease. *The Lancet*, *379* (9810), 55–70.	53.254	492	82.0
91	Grant, B. F., Saha, T. D., Ruan, W. J., Goldstein, R. B., Chou, S. P., Jung, J., ... & Hasin, D. S. (2016). Epidemiology of DSM-5 drug use disorder: Results from the National Epidemiologic Survey on Alcohol and Related Conditions–III. *JAMA Psychiatry*, *73* (1), 39–47.	16.642	491	245.5
92	Panikashvili, D., Simeonidou, C., Ben-Shabat, S., Hanuš, L., Breuer, A., Mechoulam, R., & Shohami, E. (2001). An endogenous cannabinoid (2-AG) is neuroprotective after brain injury. *Nature*, *413* (6855), 527–531.	41.577	488	28.7
93	Hall, W., & Degenhardt, L. (2009). Adverse health effects of non-medical cannabis use. *The Lancet*, *374* (9698), 1383–1391.	53.254	486	54.0
94	Okamoto, Y., Morishita, J., Tsuboi, K., Tonai, T., & Ueda, N. (2004). Molecular characterization of a phospholipase D generating anandamide and its congeners. *Journal of Biological Chemistry*, *279* (7), 5298–5305.	4.011	486	34.7
95	Di, S., Malcher-Lopes, R., Halmos, K. C., & Tasker, J. G. (2003). Nongenomic glucocorticoid inhibition via endocannabinoid release in the hypothalamus: a fast feedback mechanism. *Journal of Neuroscience*, *23* (12), 4850–4857.	5.971	486	32.4
96	Di Marzo, V., & Matias, I. (2005). Endocannabinoid control of food intake and energy balance. *Nature Neuroscience*, *8* (5), 585–589.	19.912	478	36.8
97	Linszen, D. H., Dingemans, P. M., & Lenior, M. E. (1994). Cannabis abuse and the course of recent-onset schizophrenic disorders. *Archives of General Psychiatry*, *51* (4), 273–279.	16.642	477	19.9
98	Chevaleyre, V., Takahashi, K. A., & Castillo, P. E. (2006). Endocannabinoid-mediated synaptic plasticity in the CNS. *Annual Review of Neuroscience*, *29*, 37–76.	14.675	474	39.5
99	Pertwee, R. G. (2001). Cannabinoid receptors and pain. *Progress in Neurobiology*, *63* (5), 569–611.	14.163	472	27.8
100	Hohmann, A. G., Suplita, R. L., Bolton, N. M., Neely, M. H., Fegley, D., Mangieri, R., ... & Duranti, A. (2005). An endocannabinoid mechanism for stress-induced analgesia. *Nature*, *435* (7045), 1108–1112.	41.577	469	36.1

Impact factor 2017 of *JAMA Psychiatry* is used to represent *Archives of General Psychiatry*, which was re-named as the former before. Similarly, impact factor 2017 of *FEBS Journal* is used to represent *European Journal of Biochemistry*

Adjusted citation count is total citation count divided by years since publication

### Bubble maps

**Fig. 2:**
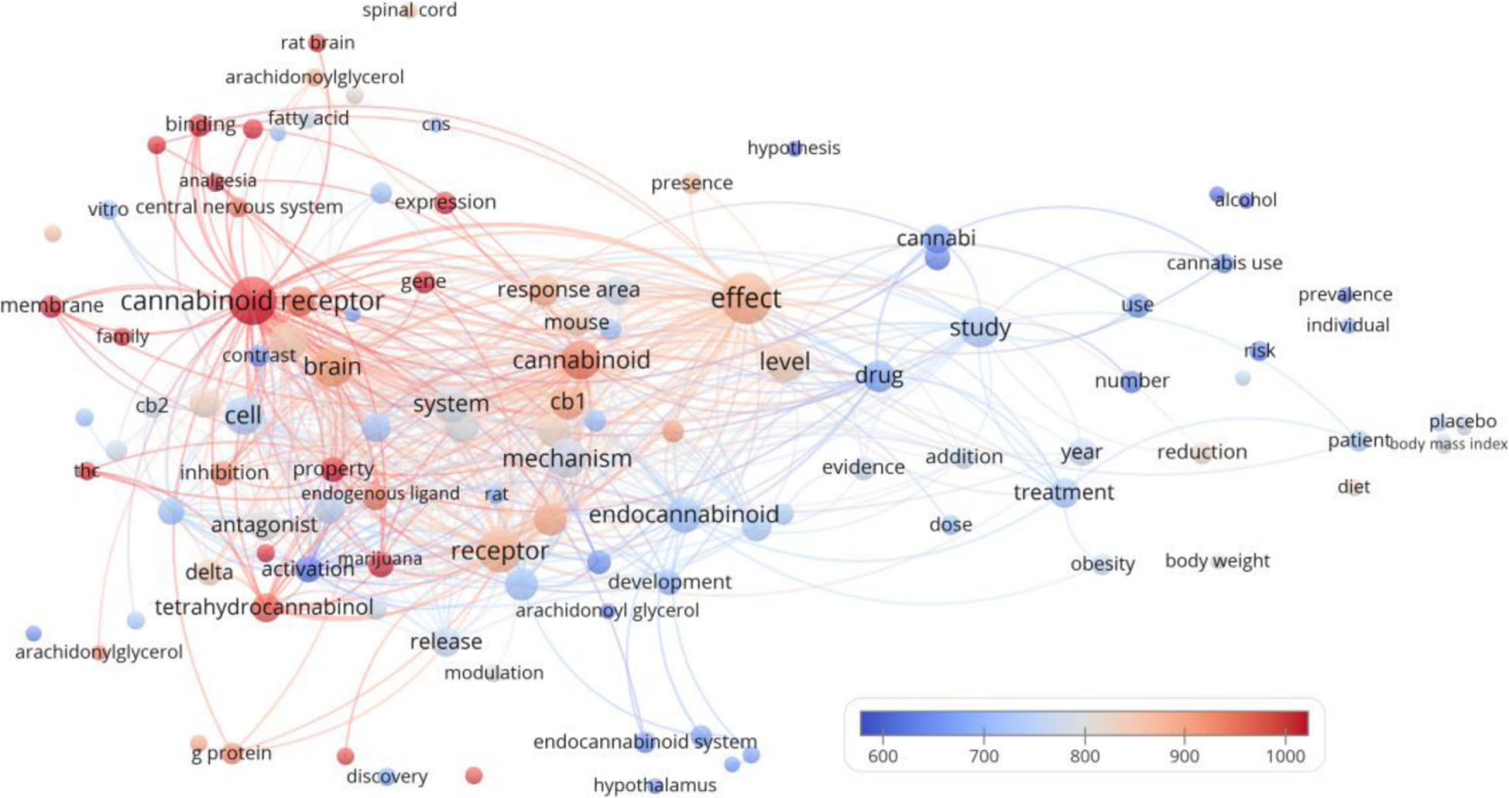
Bubble map showing words from titles and abstracts of the 100 most-cited cannabis manuscripts. Words from titles and abstracts were parsed, analyzed and visualized by VOSviewer. There were 113 terms that appeared in 5 or more manuscripts and hence were included in the map. Each bubble represents a word or phrase. The bubble size indicates its appearance frequency. The bubble color indicates the averaged citation count received by manuscripts containing the term. If two terms co-appeared in more manuscripts, the two bubbles are closer to each other. The lines indicate the 500 strongest co-appearance links between the terms

There were 113 terms that appeared in the titles and abstracts of at least 5 of the 100 manuscripts (Fig. 2). The bubble map showed that manuscripts concerning analgesia seemed to have more citations than those concerning weight loss. Moreover, tetrahydrocannabinol (THC), which also has analgesic effects, also received many citations (*n* = 16, citations per manuscript = 972.4) (Munro et al. 1993). We further examined the dataset to look for some notable terms that might appear in fewer than 5 of the manuscripts, and found that opiate was mentioned in 2 manuscripts (citations per manuscript = 748.5), whereas the non-psychoactive compounds cannabidiol (CBD) and cannabinol (CBN) were mentioned in 2 manuscripts (citations per manuscript = 598.5) and 1 manuscript (citations = 568), respectively.

**Fig. 3:**
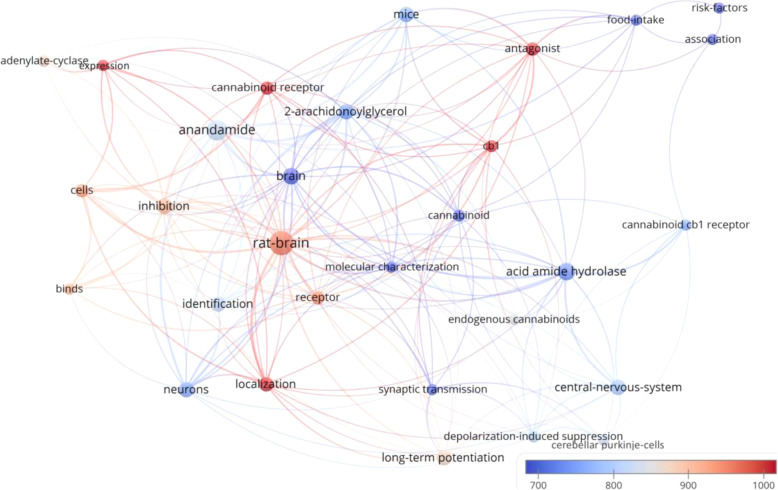
Bubble map showing keywords listed by the 100 most-cited cannabis manuscripts. Keywords added by the authors and by Web of Science (KeyWords Plus) were analyzed and visualized by VOSviewer. There were 30 keywords that appeared in 5 or more manuscripts and hence included in the map. Each bubble represents a keyword. The bubble size indicates its appearance frequency. The bubble color indicates the averaged citation count received by manuscripts containing the keyword. If two keywords co-appeared in more manuscripts, the two bubbles are closer to each other. The lines indicate the 500 strongest links between the keywords

**Fig. 4:**
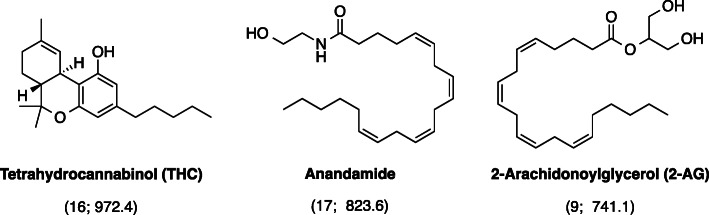
The structures of the chemicals frequently mentioned in the top 100 cannabis-related manuscripts. The number of publications and citations per publication for each chemical are provided in brackets

There were 30 keywords that appeared in at least 5 of the 100 manuscripts (Fig. 3). The bubble map of keywords showed that the rat and mouse models were frequently used (keywords: “rat brain” (*n* = 23) and “mice” (*n* = 9)). “Long-term potentiation” (n = 9), “depolarization-induced suppression” (*n* = 6), and “food intake” (*n* = 5) were frequently mentioned concepts. Long-term potentiation is considered as one of the most crucial mechanisms underlying learning and memory (Bliss and Collingridge 1993). Rat study has revealed that long-term cannabinoid treatment can undermine reference and working memory performance, and impair long-term potentiation in the hippocampus (Hill et al. 2004). In addition, endocannabinoids may have a role in modulation of anxiety and conditioned fear via long-term potentiation (Marsch et al. 2007; Marsicano et al. 2002). Meanwhile, depolarization-induced suppression of inhibition mediated via endocannabinoids / cannabinoid type 1 (CB1) receptor is a primary cortical process that enables neurons to communicates backwards across synapses to modulate their inputs, and thus contributes to multiple forms of cortical plasticity and synaptic strengthening (Kreitzer and Regehr 2001; Ohno-Shosaku et al. 2001; Wilson and Nicoll 2001). With respect of food intake, endocannabinoids in the hypothalamus might conditionally activate CB1 receptors to maintain and regulate food intake together with leptin (Di Marzo et al. 2001). Findings from clinical trial have demonstrated that the intake of CB1 receptor blocker, rimonabant, together with hypocaloric diet could lead to significant decrease in body weight and risk of having cardiovascular disease (Van Gaal et al. 2005). However, rimonabant was withdrawn for treatment due to its adverse effects such as causing mood swings and suicide (Christensen et al. 2007). Research is still ongoing for food intake regulation and it is proposed that peripherally restricted CB1 receptor blockers may be therapeutic in the future (Simon and Cota 2017). Moreover, the CB1 receptor was frequently the focus of research, with frequent recurrence of keywords: “CB1” (*n* = 6), “cannabinoid CB1 receptor” (*n* = 5), and anandamide (*n* = 17, citations per manuscript = 823.6), its famous agonist reported by the manuscript with the highest citation count (Devane et al. 1992). When appearances in titles, abstracts and keywords are considered together, CB1 appears in 23 manuscripts, whereas CB2 emerged in 10 manuscripts. One important function of CB2 receptors is modulation of mast cell activation and thus inflammation via agonist binding (Facci et al. 1995). Thus, it seems that the CB1 receptor was studied by more of the top 100 papers in comparison to the CB2 receptor. Another frequently mentioned chemical was 2-arachidonoylglycerol (*n* = 9, citations per manuscript = 747.1), an endocannabinoid that mediates analgesia, hypothermia, hypomotility and modulates long-term potentiation (Hanuš et al. 2001; Long et al. 2009; Stella et al. 1997). The structures of these frequently mentioned chemicals are listed in Fig. 4.

### General thoughts on existing evidence

The literature analysis has revealed that there were many rat and mice studies. The primary limitation of murine studies is they do not accurately mirror the wide spectrum of variability in the treatment response of the most human subjects tested with cannabis-based medicine. Reactions and effects may vary significantly due to for example genetic predispositions causing varying amounts of endocannabinoid receptors. Moreover, a complex multi-molecule mentality may be more suitable for understanding the cannabinoid compound class than a small molecule mentality, due to the limited number of over 10 active compounds that work in unison to create successful treatment. For example, without some amounts of THC, cannabidiol (CBD) becomes relatively inert as an anti-inflammatory agent (Aso et al. 2015). THC and CBD can also work in synergism for other therapeutic purposes such as neuroprotection and managing neoplasms (Burstein 2015; Russo and Guy 2006). This is why traditional pharmaceuticals-mentality sometimes may struggle to comprehend cannabis-based (as well as in general plant medicine-based) effects because isolating specific compounds from medicinal plants often results in poorer results or requiring significantly higher doses.

### Study limitations

Our study was designed such that manuscripts were collated from a single database only, the WoS Core Collection, so that we might have missed some relevant manuscripts in the literature. This was decided because citation numbers for each manuscript differ across different databases, and merging data from multiple databases is a complicated yet controversial challenge. We chose to focus on WoS Core Collection for our study because it is hosted by Clarivate Analytics, the same company that releases the journal impact factors and thus represents the most established authority in the area of research evaluation. This also allowed a better exploration of relationship between citation counts and journal impact factors. Finally, it should be noticed that 100 analyzed publications constitute only a very small percentage of the entire literature on the topic. Thus, the publications might not represent the complete literature but rather reflect the most influential research of this scientific area.

### Cannabis- and cannabinoids-related treatments: what is new?

More than two decades have passed until enormous changes have occurred in legal situation and social policies in regards to the use of cannabis for medical and other purposes (Corroon Jr et al. 2017). Today, there are around 30 countries worldwide that have legalized medical cannabis or cannabis-derived products for certain uses, including the United States (33 states so far plus the District of Columbia), Australia, Argentina, Canada, Chile, Israel, Mexico, Switzerland, Turkey, Uruguay, 15 European Union countries, and others. However, there are very strict guidelines regulating the medical use of cannabis-derived pharmaceuticals. It should be noticed, that some EU countries such as the Netherlands, Poland, Norway, Germany, Italy, and others are leaders in cannabis legalization so far, i.e., legalized access for the needs of patients with medical conditions. In other EU countries such as Spain, France, and Slovenia, the use of cannabis-derived drugs for some afflictions is permitted. Meanwhile, Uruguay and Canada are the only countries that legalized the sale and consumption of cannabis for recreational use. In the United States, 14 states have more restrictive laws limiting THC content, while ten states and D. C. have legalized the recreational use of cannabis. In the United States, the cannabidiol (CBD) containing medicine Epidiolex was recently approved by the FDA as the first prescription drug available for the treatment of rare diseases such as Dravet syndrome (also known as severe myoclonic epilepsy of infancy, SMEI) or Lennox-Gastaut syndrome (LGS) (Rubin 2018). In addition, the FDA-approved drugs Marinol, Syndros and Cesamet represent synthetic cannabinoids, which structures are similar to that of THC (Rubin 2018). All these medicines are used to treat nausea and vomiting caused by cancer chemotherapy, whereas the first two are also used as appetizer in the treatment of patients with AIDS (Rubin 2018).

## Conclusions

A bibliometric analysis was conducted to identify the 100 most cited endocannabinoid, cannabis and cannabinoid-related manuscripts. The top-ranked manuscripts among the 100 concerned analgesia, weight loss, long-term potentiation, depolarization-induced suppression of inhibition, and other topics. The CB1 receptor was studied by more of the top 100 papers in comparison to the CB2 receptor. The most frequently mentioned chemicals in these publications were 2-arachidonoylglycerol, tetrahydrocannabinol, and anandamide. Together, these manuscripts comprise the most highly cited literature body on the topic, literally the molecular neuroscience at its “high”.

## Abbreviations

CB1: Cannabinoid type 1
CB2: Cannabinoid type 2
CBD: Cannabidiol
CBN: Cannabinol
FDA: Food and Drug Administration
THC: Tetrahydrocannabinol
WoS: Web of Science
